# Acute Granulomatous Appendicitis and Lower Gastrointestinal Bleeding as the Presenting Features of Crohn's Disease

**DOI:** 10.7759/cureus.5793

**Published:** 2019-09-28

**Authors:** Ariel H Park, Carlos E Ramos, Vladimir Neychev

**Affiliations:** 1 Medicine, University of Central Florida College of Medicine, Orlando, USA; 2 Pathology, Health Care Corporation of America, Longwood, USA; 3 Surgery, University of Central Florida College of Medicine, Orlando, USA

**Keywords:** crohn's disease, granulomatous appendicitis, appendiceal crohn's disease

## Abstract

A 46-year-old woman with a history of hemorrhoids presented with right-sided abdominal pain and rectal bleeding. Preoperative imaging and intraoperative observations were suggestive of acute appendicitis. The surgical pathology of removed appendix was consistent with granulomatous appendicitis. The patient was evaluated again in two months due to persistent hematochezia and new onset of left lower quadrant abdominal pain. A diagnostic colonoscopy revealed mildly edematous mucosa in the descending colon, sigmoid colon, and rectum, and a rectal biopsy revealed patchy chronic proctitis. The biopsy of anal canal mucosa showed acute and chronic granulomatous inflammation. Based on her clinical presentation and pathology results, the diagnosis of chronic active proctitis secondary to Crohn’s disease (CD) was made. There is a debate on necessity of follow-up on patients with appendiceal CD after appendectomy as previously reported appendiceal CD usually follows a benign course post-appendectomy. However, our patient’s case progressed from granulomatous appendicitis to CD with severe GI bleeding and proctitis within only two months post-appendectomy. A high index of suspicion is needed in patients with a history of granulomatous appendicitis and lower GI bleeding to ensure prompt diagnosis and timely treatment.

## Introduction

Crohn’s disease (CD) is a chronic relapsing inflammatory bowel disease, which is characterized by a transmural granulomatous inflammation involving any part of the gastrointestinal tract but most commonly the ileum, colon, or both [1]. Meyerding first described a case of isolated CD of the appendix in 1953 and since then, a number of cases of appendiceal CD have been reported in the literature [2,3]. The incidence of appendicitis with granulomatous reaction varies from 0.1% to 2.0% of appendectomy cases, and upto 50% of specimens resected in cases of CD showed appendiceal involvement [3-5]. It has been noted that primary CD of the appendix has a more favorable clinical outcome compared to CD arising in small or large bowel with long post-surgical remissions and a recurrence rate of 8% to 10% [6-8]. We report a case of CD with initial presentation of granulomatous appendicitis which recurred within two months post-appendectomy. 

## Case presentation

**Figure 1:**
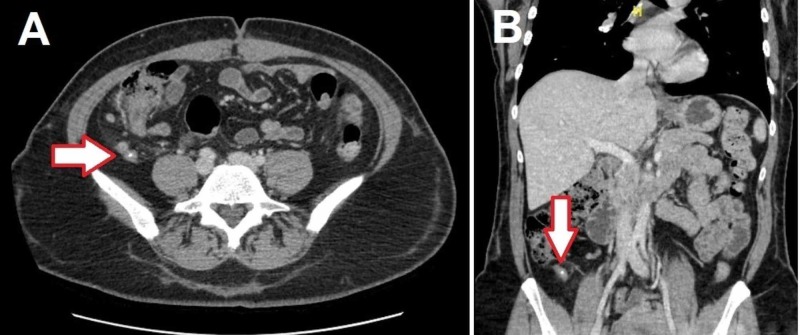
Computed tomography of the abdomen and pelvis A, Axial, and B, coronal images of the abdomen and pelvis with findings suggestive of an acute appendicitis with an appendicolith (arrows).

**Figure 2:**
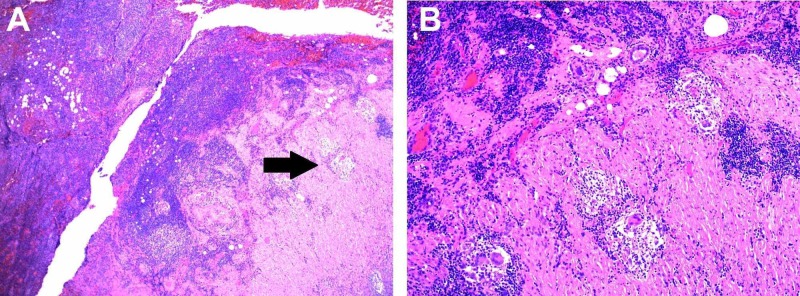
Surgical H&E histopathology A, Low power of the acute granulomatous appendicitis with arrow pointing at the granuloma. B, High power of the granulomatous appendicitis.

A 46-year-old female with a history of internal and external hemorrhoids initially presented to the emergency room with rectal bleeding. The patient noticed intermittent bright red blood per rectum with some pain for the past several months, which she suspected to be a hemorrhoidal bleeding. For the last three days, she noticed worsening rectal bleeding and mucus discharge. The patient also complained of right-sided abdominal pain, which resembled her perimenstrual symptoms but had become constant for the past three weeks. Her vital signs were within normal limits, and the lab values were unremarkable with white blood cell of 9.8 x 109/L and hemoglobin of 11.7 g/dL. Physical examination showed tenderness near the right iliac fossa and external hemorrhoids without any signs of bleeding. Her CT abdomen revealed acute appendicitis with appendicolith (Figure 1A, 1B), and thus, the patient underwent laparoscopic appendectomy. During the surgery, phlegmonous appendicitis and local peritonitis were encountered, and the inspection of the abdominal cavity revealed no other abnormalities. The surgical pathology results were consistent with acute and chronic granulomatous appendicitis (Figure 2A, 2B). No fungi, caseous necrosis, or acid-fast bacilli were reported.

**Figure 3:**
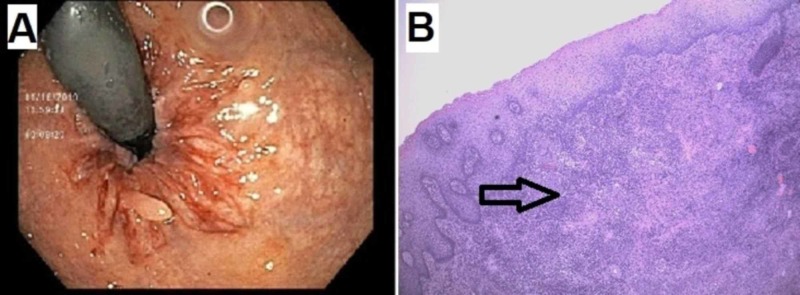
Colonoscopy and anal mucosa biopsy findings A, Retroflexed view revealed no large internal hemorrhoids. Squamous mucosa below dentate line appeared to be quite erythematous and friable on contact. B, Biopsy of anal canal mucosa showed acute and chronic granulomatous inflammation (arrow).

Two months post-appendectomy, the patient was evaluated again for persistent hematochezia as well as new onset of left lower quadrant abdominal pain. The bleeding was presumed to be secondary to hemorrhoids, and the patient underwent a diagnostic colonoscopy prior to possible hemorrhoidectomy. The colonoscopy revealed mildly edematous mucosa in the descending colon, sigmoid colon, and rectum. The squamous mucosa below dentate line also appeared to be erythematous and friable with easy oozing on contact (Figure 3A). No large hemorrhoids were noticed on retroflexed view of the anorectal area. Random biopsies taken from the descending and sigmoid colon during colonoscopy showed benign colonic mucosa. However, the rectal biopsy revealed patchy chronic proctitis without adenomatous, dysplastic, or malignant changes. Examination under anesthesia with biopsy of anal canal mucosa showed acute and chronic granulomatous inflammation (Figure 3B). There was no evidence of acid-fast bacilli or herpes simplex virus (or viral). Grade 2 internal hemorrhoids and external skin tags suggestive of prior external hemorrhoids were also noted during the procedures.

Based on her clinical presentation and pathology results from her appendectomy, colonoscopy, and anal canal biopsy, the diagnosis of chronic active proctitis secondary to CD was made. The patient was started on mesalamine 4.8 g daily and she was symptom free at six months follow-up.

## Discussion

When regional ileitis, which is now known as CD, was first described by Dr. Burrill B. Crohn, the inflammation was thought to never transcend the limit of Bauhin’s valve nor to involve the appendix [9]. However, subsequent studies revealed that up to 50% of CD involve appendix [3]. Although isolated granulomatous appendicitis is considered an uncommon presentation of CD, an increasing number of cases has been reported in the literature [10].

Various causes of granulomatous appendicitis include infections by fungi, *Yersinia pseudotuberculosis*, which account for 25% of cases, and *Mycobacterium tuberculosis*, foreign bodies, and systemic conditions such as CD and sarcoidosis [11-13]. It can be difficult to distinguish lesions of different etiologies merely based on histological examination, especially between idiopathic granulomatous appendicitis and early CD with only the appendix involved [14]. Incorporating clinical presentation, past medical history, histological and serological results, and long-term follow-up are all important in making the final diagnosis. A wide range of differential diagnosis of CD makes it difficult for early detection and diagnosis. Differential diagnosis includes infections, diverticulitis, ulcerative colitis, Behçet’s syndrome, sarcoidosis, celiac disease, bile acid loss, overuse of nonsteroidal anti-inflammatory drugs, colorectal cancer, irritable bowel syndrome, and ischemic colitis [15]. 

Although the typical clinical manifestations of CD are variable, more than 90% of patients present with diarrhea and abdominal pain, 85% with greater than 5 lb weight loss, 56% with fever, and positive stool tests for occult blood in 27% of patients [16]. Although microscopic bleeding is frequently seen in CD patients through positive guaiac or immunochemical test, gross bleeding is rare [17]. Given that it takes 35 months on average from the onset of symptoms to diagnosis of CD, it is possible that our patient had been experiencing the rare symptom of GI bleeding without more common symptoms alongside [16]. However, her history of hemorrhoids makes it difficult to discern whether her rectal bleeding prior to her appendectomy was truly due to underlying CD. As our patient noticed mucus in her rectal bleeding and worsening abdominal pain three days and three weeks prior to appendectomy, respectively, she may have developed symptoms of CD shortly before the surgery.

Previously reported appendiceal CD usually follow a benign course post-appendectomy. Appendiceal CD seems to have a much better prognosis and lower recurrence rate than those of CD developing from small or large bowel [6,18]. Vanek et al. reported a recurrence rate of 8% occurring 19 months on average since diagnosis [6]. More recent cases in the literature report no evidence of disease recurrence in their patients [3,19,20]. However, our patient’s case progressed from granulomatous appendicitis to CD with severe GI bleeding and proctitis within only two months post-appendectomy. Her long history of hemorrhoids and occasional hemorrhoidal bleeding could lead to premature dismissal of her recurring hematochezia. However, her initial presentation with abdominal pain, rectal bleeding, and granulomatous changes of her appendix prompted her physicians to follow-up on the patient closely and to obtain colonoscopy and biopsy to ensure her health.

## Conclusions

There is a debate on necessity of follow-up on patients with appendiceal CD after appendectomy. Some believe that appendectomy is curative in most cases, whereas others recommend a follow-up of five years. Given that our patient developed recurrence relatively quickly, we believe a high index of suspicion is needed in patients with a history of granulomatous appendicitis and lower GI bleeding. A close follow-up as well as thorough colonoscopy with multiple random biopsies and those of the suspicious areas of the GI tract will ensure prompt diagnosis and timely treatment.

## References

[1] Thia KT, Sandborn WJ, Harmsen WS, Zinsmeister AR, Loftus EV, Jr Jr (2010). Risk factors associated with progression to intestinal complications of Crohn's disease in a population-based cohort. Gastroenterology.

[2] Meyerding EV, Bertram HF (1953). Nonspecific granulomatous inflammation (Crohn's disease) of the appendix: a case report. Surgery.

[3] Bischoff A, Gupta A, D'Mello S (2010). Crohn's disease limited to the appendix: a case report in a pediatric patient. Pediatr Surg Int.

[4] AbdullGaffar B (2010). Granulomatous diseases and granulomas of the appendix. Int J Surg Pathol.

[5] Nugent FW: Editorial (1976). Crohn's disease of the appendix. Am J Gastroenterol.

[6] Yang SS, Gibson P, McCaughey RS, Arcari FA, Bernstein J (1979). Primary Crohn's disease of the appendix: report of 14 cases and review of the literature. Ann Surg.

[7] Vanek VW, Spirtos G, Awad M, Badjatia N, Bernat D (1988). Isolated Crohn's disease of the appendix. Two case reports and a review of the literature. Arch Surg.

[8] Agha FP, Ghahremani GG, Panella JS, Kaufman MW (1987). Appendicitis as the initial manifestation of Crohn's disease: radiologic features and prognosis. AJR Am J Roentgenol.

[9] Crohn BB, Ginzburg L, Oppenheimer GD (1932). Regional ileitis: a pathologic and clinical entity. JAMA.

[10] Richards ML, Aberger FJ, Landercasper J (1997). Granulomatous appendicitis: Crohn's disease, atypical Crohn's or not Crohn's at all?. J Am Coll Surg.

[11] Tucker ON, Healy V, Jeffers M, Keane FB (2003). Granulomatous appendicitis. Surgeon.

[12] Bronner MP (2004). Granulomatous appendicitis and the appendix in idiopathic inflammatory bowel disease. Semin Diagn Pathol.

[13] Brown I, Kumarasinghe MP (2018). Granulomas in the gastrointestinal tract: deciphering the Pandora's box. Virchows Arch.

[14] Ho P, Law WL, Choy C, Chan GS, Chu KW (2003). Granulomatous appendicitis progressing to Crohn's disease with bleeding complication. ANZ J Surg.

[15] Cummings JR, Keshav S, Travis SP (2008). Medical management of Crohn's disease. BMJ.

[16] Mekhjian HS, Switz DM, Melnyk CS, Rankin GB, Brooks RK (1979). Clinical features and natural history of Crohn's disease. Gastroenterology.

[17] Joachim G (1986). An update on inflammatory bowel disease. AAOHN J.

[18] Ariel I, Vinograd I, Hershlag A (1986). Crohn's disease isolated to the appendix: truths and fallacies. Hum Pathol.

[19] Han H, Kim H, Rehman A, Jang SM, Paik SS (2014). Appendiceal Crohn's disease clinically presenting as acute appendicitis. World J Clin Cases.

[20] Yokota S, Togashi K, Kasahara N (2010). Crohn's disease confined to the appendix. Gastrointest Endosc.

